# Age-dependent effects of microglial inhibition *in vivo* on Alzheimer’s disease neuropathology using bioactive-conjugated iron oxide nanoparticles

**DOI:** 10.1186/1477-3155-11-32

**Published:** 2013-09-23

**Authors:** Micaela Glat, Hadas Skaat, Noa Menkes-Caspi, Shlomo Margel, Edward A Stern

**Affiliations:** 1 Gonda Multidisciplinary Brain Research Center, Bar-Ilan University, Ramat-Gan 52900, Israel; 2 Department of Chemistry, Bar-Ilan Institute of Nanotechnology and Advanced Materials, Ramat-Gan 52900, Israel; 3 MassGeneral Institute of Neurodegenerative Disease, Massachusetts Genral Hospital and Harvard Medical School, Charlestown, MA 02129, USA

**Keywords:** Tau, Microglia, Tangle, Fibrin γ^377-395^ peptide, Iron oxide nanoparticles

## Abstract

**Background:**

Tau dysfunction is believed to be the primary cause of neurodegenerative disorders referred to as tauopathies, including Alzheimer’s disease, Pick’s disease, frontotemporal dementia and Parkinsonism. The role of microglial cells in the pathogenesis of tauopathies is still unclear. The activation of microglial cells has been correlated with neuroprotective effects through the release of neurotrophic factors and through clearance of cell debris and phagocytosis of cells with intracellular inclusions. In contrast, microglial activation has also been linked with chronic neuroinflammation contributing to the development of neurodegenerative diseases such as tauopathies. Microglial activation has been recently reported to precede tangle formation and the attenuation of tau pathology occurs after immunosuppression of transgenic mice.

**Methods:**

Here we report the specific inhibition of microglial cells in rTg4510 tau-mutant mice by using fibrin γ^377-395^ peptide conjugated to iron oxide (γ-Fe_2_O_3_) nanoparticles of 21 ± 3.5 nm diameter.

**Results:**

Stabilization of the peptide by its covalent conjugation to the γ-Fe_2_O_3_ nanoparticles significantly decreased the number of the microglial cells compared to the same concentration of the free peptide. The specific microglial inhibition induces different effects on tau pathology in an age dependent manner. The reduction of activation of microglial cells at an early age increases the number of neurons with hyperphosphorylated tau in transgenic mice. In contrast, reduction of activation of microglial cells reduced the severity of the tau pathology in older mice. The number of neurons with hyperphosphorylated tau and the number of neurons with tangles are reduced than those in animals not receiving the fibrin γ^377-395^ peptide-nanoparticle conjugate.

**Conclusions:**

These results demonstrate a differential effect of microglial activity on tau pathology using the fibrin γ^377-395^ peptide-nanoparticle conjugate, depending on age and/or stage of the neuropathological accumulation and aggregation.

## Introduction

Tau protein is present in phosphorylated and aggregated form in Alzheimer’s disease (AD) and in a group of neurodegenerative disorders collectively termed tauopathies [1].

Tau is a microtubule-associated protein (MAP) that under normal physiological conditions is involved in microtubule assembly and stabilization [2,3]. In the normal adult human brain, six isoforms of tau are produced from a single gene by alternative mRNA splicing in exons 2, 3, and 10 of the tau gene located on chromosome 17 [4,5]. Tau occurs mainly in axons, whereas another MAP protein, MAP2, is localized to the somatodendritic compartment [6].

Tauopathies are characterized by an abnormal hyperphosphorylation of the tau protein in sites not normally phosphorylated and later assembled into neurofibrillary tangles (NFTs) in neuronal cell bodies and sometimes in glial cells [7]. Early hyperphosphorylation is facilitated by kinases such as glycogen synthase kinase 3 (GSK3), cyclin-dependent kinase 5 (cdk5) and c-Jun N-terminal kinase (JNK) [8,9]. Hyperphosphorylation and the formation of NFTs create conditions in which the tau protein is unable to bind with microtubules, producing impairments in axonal trafficking and profound effects on the function and viability of neurons, contributing to synaptic dysfunction and neurodegeneration [10-12].

Microglial cells are the resident immune cells of the CNS [13,14]. Under physiological conditions, residential microglial cells are quiescent and scattered throughout the CNS [15], and are characterized by a small cell body and a ramified morphology. Occasionally, microglia will become moderately activated in order to play the classic role as “scavengers” for the maintenance and restoration of the CNS. They begin to proliferate, changing their morphology into an amoeboid shape and phagocytose cells that are pathologically damaged or developmentally unnecessary [16]. These functions of microglia are controlled by communication with cytokines, chemokines, trophic factors, and other neuromodulating molecules among neurons, astrocytes and microglia [17].

Several studies have shown that microglial cells are beneficial for the proper function of the CNS through phagocytosis of cell debris [18-20]. It has been suggested that this phagocytic activity plays a fundamental role in facilitating reorganization of neuronal circuits and triggering repair in neurodegenerative diseases. Furthermore, it was shown that insufficient clearance by microglia, prevalent in tau pathology and declining with age, is associated with an inadequate regenerative response [21]. Activated microglial cells have also been reported to possess neuroprotective/neurotrophic effects *in vitro*[22]. Activated microglia could secrete some neurotrophic factors such as NGF, NT-3 and BDNF which have been demonstrated to be neuroprotective [23]. However, other studies have shown that inflammation plays a key role in the progression of neurodegenerative diseases such as AD. In an animal model of tauopathies, early microglial activation was associated with loss of synapses preceding tangle formation, suggesting that microglial activation is a cause of neuronal degeneration rather than a consequence of it [24-26]. Therefore, controversy exists about the role microglial function plays in the development of the progression of neurodegenerative diseases, driving the need for a more precise method of testing microglial function. To do so, we have utilized a specific inhibitor of microglial cells, the fibrinogen-derived γ^377-395^ peptide, for the inhibition of microglial cells at various stages of the tauopathy disease.

The fibrinogen-derived γ^377-395^ peptide has been recently reported as a specific inhibitor of microglial activation via the MAC-1 receptor (α_M_β_2_, CD11b/CD18) [27,28]. The main disadvantage of fibrin γ^377-395^ peptide is its short *in vivo* half-life time due to rapid enzymatic degradation, leading to loss of biological activity and functions. It requires, therefore, frequent direct injections of the peptide in order to maintain its bioavailability necessary for microglia inactivation. However, repeated direct intracranial administration may lead to undesirable systematic effects and toxicity. To obtain a steady administration over-time, peptides are adsorbed onto, or encapsulated within, nano-materials to protect their stability and biological activity in a sustained and controllable manner [29].

Magnetic iron oxide (maghemite, γ-Fe_2_O_3_) nanoparticles are particularly promising due to their high surface area to volume ratio, magnetic properties, biocompatibility, relative non-toxicity, and biodegradability. The use of iron oxide magnetic nanoparticles for various biomedical applications, e.g., hyperthermia, diagnosis, cell-labeling and sorting, DNA separation, MRI contrast agents and drug delivery, have already been demonstrated [30-35].

In this study, we present a novel approach for specific inhibition of microglial cells in rTg4510 tau-transgenic mice by using fibrin γ^377-395^ peptide-conjugated γ-Fe_2_O_3_ nanoparticles of 21 ± 3.5 nm diameter. The stabilization of the peptide by its conjugation to these nanoparticles significantly decreased the number of the microglial cells compared to the same concentration of the free peptide. Furthermore, the specific inhibition of microglial cells, attained using the γ^377-395^ peptide-conjugated γ-Fe_2_O_3_ nanoparticles, was found to have a dual effect on tau pathology depending on the age of the mice used in the study.

## Results

### Specific inhibition of microglial cells using fibrin γ^377-395^ peptide-conjugated γ-Fe_2_O_3_ nanoparticles

**Figure 1:**
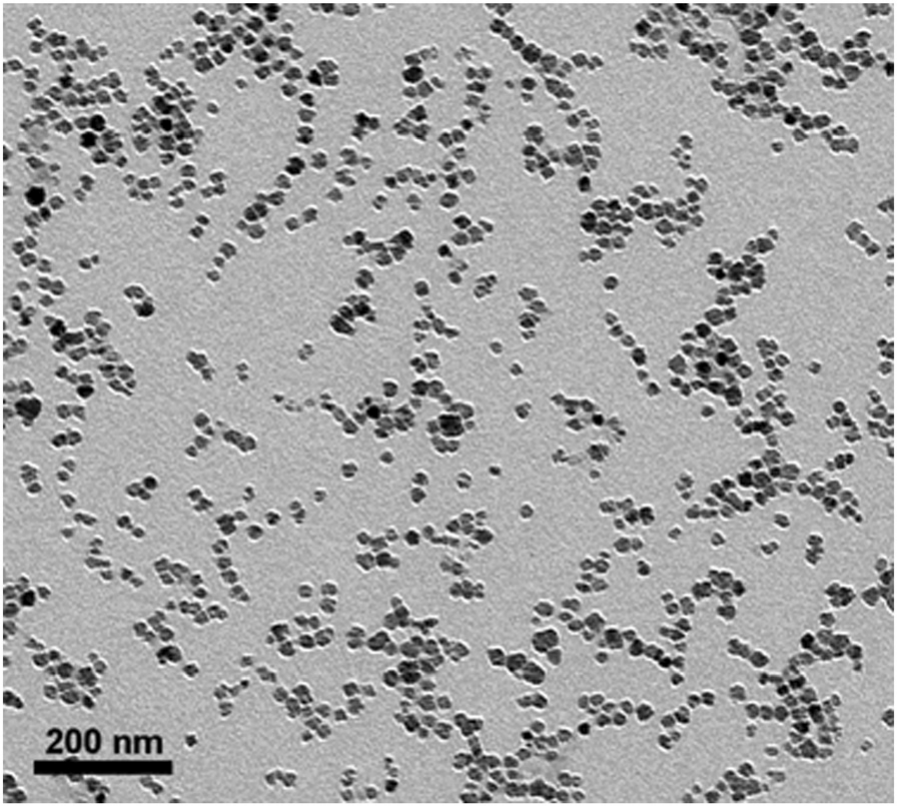
**TEM image of the fibrin γ**^**377-395 **^**peptide-conjugated γ-Fe**_**2**_**O**_**3 **_**nanoparticles.** The transmission electron microscope (TEM) image of the fibrin γ^377-395^ peptide-conjugated γ-Fe_2_O_3_ nanoparticles demonstrates that these nanoparticles are stable against agglomeration and possess a diameter of 21 ± 3.5 nm.

**Figure 2:**
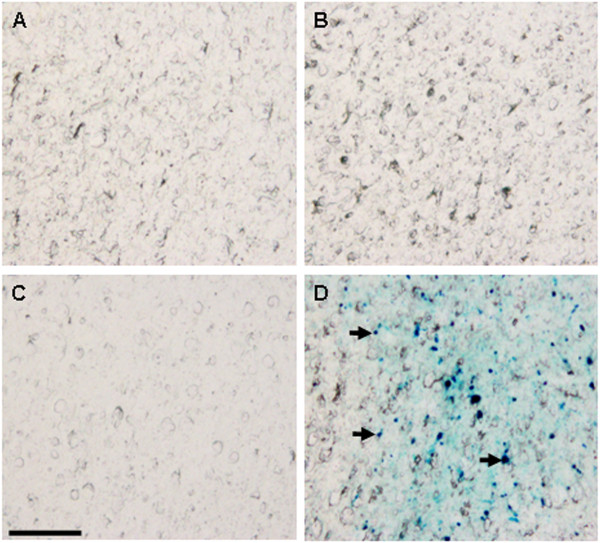
**Localization of the fibrin γ**^**377-395 **^**peptide-conjugated γ-Fe**_**2**_**O**_**3 **_**nanoparticles in the frontal cortex.** The γ-Fe_2_O_3_ nanoparticles were stained with potassium ferocyanide (blue). No staining was seen in the hemisphere injected with saline **(A, C)** and free fibrin γ^377-395^ peptide **(B)**. Positive staining **(D)** (arrows) indicates the presence of the nanoparticles. Scale bar: 50 μm.

The fibrin derived γ^377-395^ peptide has been shown to specifically inhibit microglial activity *in vivo*[28] and was therefore chosen to facilitate the specific inhibition of microglial activity. This peptide, however, possesses a short half-life when administered in saline solution and needs to be administered constantly as a result. To counteract this issue, we have conjugated the peptide to γ-Fe_2_O_3_ nanoparticles. The transmission electron microscope (TEM) image of the fibrin γ^377-395^ peptide-conjugated γ-Fe_2_O_3_ nanoparticles shown in Figure 1 demonstrates that these nanoparticles are stable against agglomeration and possess a diameter of 21 ± 3.5 nm. These nanoparticles have been shown to enhance efficacy of delivery of bioactive material and to provide protection against biodegradation [36]. Fibrin γ^377-395^ peptide, once conjugated to the nanoparticles, is retained in the site of injection and does not disperse by diffusion. This was confirmed 30 days post intracranial injection, using potassium ferrocyanide staining, which gave a positive staining with brains treated with the conjugated peptide, whereas no positive staining was seen in brain hemispheres injected with saline or free fibrin γ^377-395^ peptide (Figure 2).

**Figure 3:**
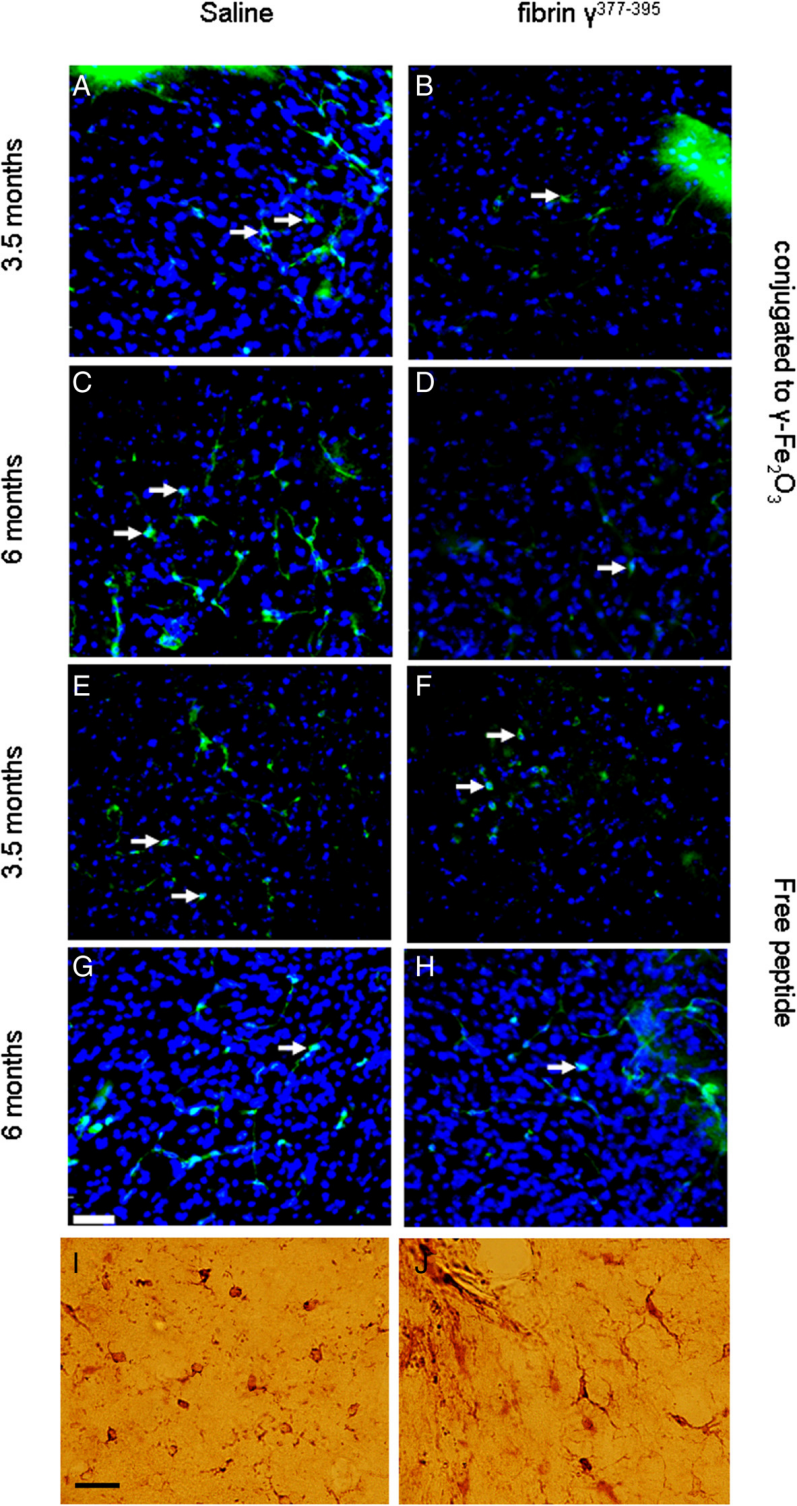
**Fibrin γ**^**377-395 **^**peptide-conjugated γ-Fe**_**2**_**O**_**3 **_**nanoparticles decreased the number of activated microglia.** rTg4510 mice at ages 3.5 **(A, B, E, F)** and 6 months **(C, D, G, H)** were injected given a single intracranial injection unilaterally with saline (left column) or fibrin γ^377-395^ peptide-conjugated γ-Fe_2_O_3_ nanoparticles **(B, D)** or free fibrin γ^377-395^ peptide **(F, H)** and were sacrificed after 30 days. Activated microglial cells were stained with the lectin IB4 (white arrows). DAPI was used for nuclear staining. Statistical analysis (see Figure 3) revealed a significant reduction in the number of activated microglia following injection with fibrin γ^377-395^ peptide-conjugated γ-Fe_2_O_3_ nanoparticles (compare **B** and **D** with **A** and **C**). Scale bar: 50 μm. **I, J;** Iba-1 staining demonstrates resting and activated microglia. **I**: saline injection; **J**: following injection with fibrin γ^377-395^ peptide-conjugated γ-Fe_2_O_3_ nanoparticles. The differences in shape of the microglia are clearly seen. Scale bar: 25 μm.

**Figure 4:**
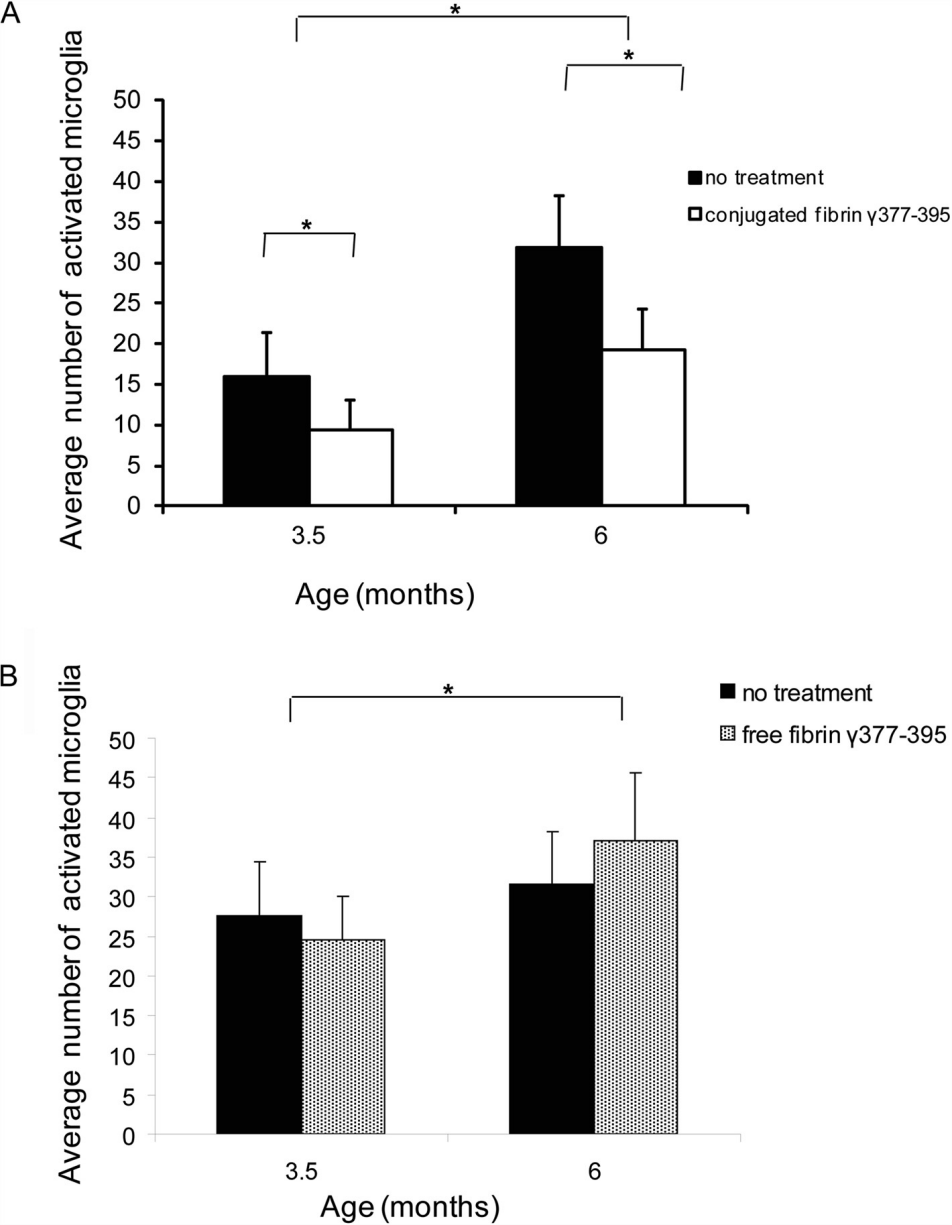
**The effect of fibrin γ**^**377-395 **^**peptide-conjugated γ-Fe**_**2**_**O**_**3 **_**nanoparticles on the number of activated microglia.** Statistical analysis was performed using 2-way ANOVA followed by Tukey multiple comparison test. Area count of the stained microglia for IB4 showed a significant decrease in the number of activated microglia at both ages when fibrin γ^377-395^ conjugated to nanoparticles was injected compared to saline **(A)** (at age 3.5 months: n = 5 mice, 171 areas; at age 6 months: n = 6, 108 areas). Although there was a significant effect for age, no significant differences were found between free fibrin γ^377-395^ and saline injections **(B)** (at age 3.5 months: n = 2 mice, 20 areas; at age 6 months: n = 3, 38 areas).

To determine the effect of fibrin γ^377-395^ peptide on activation of microglial cells, we measured the average numbers of activated microglial cells per area in animals injected with saline versus fibrin γ^377-395^ peptide-conjugated γ-Fe_2_O_3_ nanoparticles, as well as free fibrin γ^377-395^ peptide. We were unable to determine the specificity of fibrin γ^377-395^ peptide-conjugated γ-Fe_2_O_3_ nanoparticles to the microglia. Figure 3 shows examples of activated microglia cells using IB4, a lectin which stains activated microglia. The data were analyzed with a 2-way analysis of variance (ANOVA). The fibrin γ^377-395^ peptide, when conjugated to the nanoparticles, significantly reduced the number of activated microglia (white arrows) in the site of injection compared to saline (Figure 3, top 2 rows), (F = 41.01; (degrees of freedom (df) = 1,274; p ≤ 0.05). An effect of age was also found (F = 74.89; df = 1,274; p ≤ 0.05). In addition, we also found an interaction effect between age and treatment (F = 4.25; df = 1,274; p ≤ 0.05). These results are summarized in the graphs in Figure 4A. Whereas in both the saline-injected and fibrin γ^377-395^ peptide-injected animals, the number of activated microglia increased with age, the rate of increase was reduced in the fibrin γ^377-395^ peptide-injected animals, indicating that the conjugated fibrin γ^377-395^ peptides reduces the rate of activation of microglial cells. The γ-Fe_2_O_3_ nanoparticles have been shown to be an effective mechanism of delivery for peptides in the mammalian brain [37,38]. The nanoparticles alone showed no effects, and no neurotoxicity effects of the nanoparticles were observed [36-41]. In addition to staining for activated microglia exclusively, we also stained sections with ionized calcium-binding adapter molecule-1 (Iba-1) a marker for all microglia. The results of this are shown in Figure 3I and 3J, where it is seen that the percentage of amoeboid cells is higher in the hemisphere injected with fibrin γ^377-395^ peptide.

The results for injection of the unconjugated peptide revealed a different pattern: In contrast to the conjugated peptide, while the number of activated microglia increased with age, no significant changes were found between the saline and unconjugated peptide-injected conditions (Figure 3, bottom rows). These results are summarized in Figure 4B. While a significant difference was found for age (F = 6.56; df = 1,54; p ≤ 0.05), no significant differences in the number of activated microglial cells when compared with saline controls (F = 0.002; df = 1,54; NS; No significant interaction was found between age and treatment: F = 3.89, df = 1,54; NS). We interpret these results to mean that the unconjugated peptide was ineffective at reducing the rate of activation of microglial cells.

### Effects of microglial inhibition on hypersphosphorylated tau and NFTs

**Figure 5:**
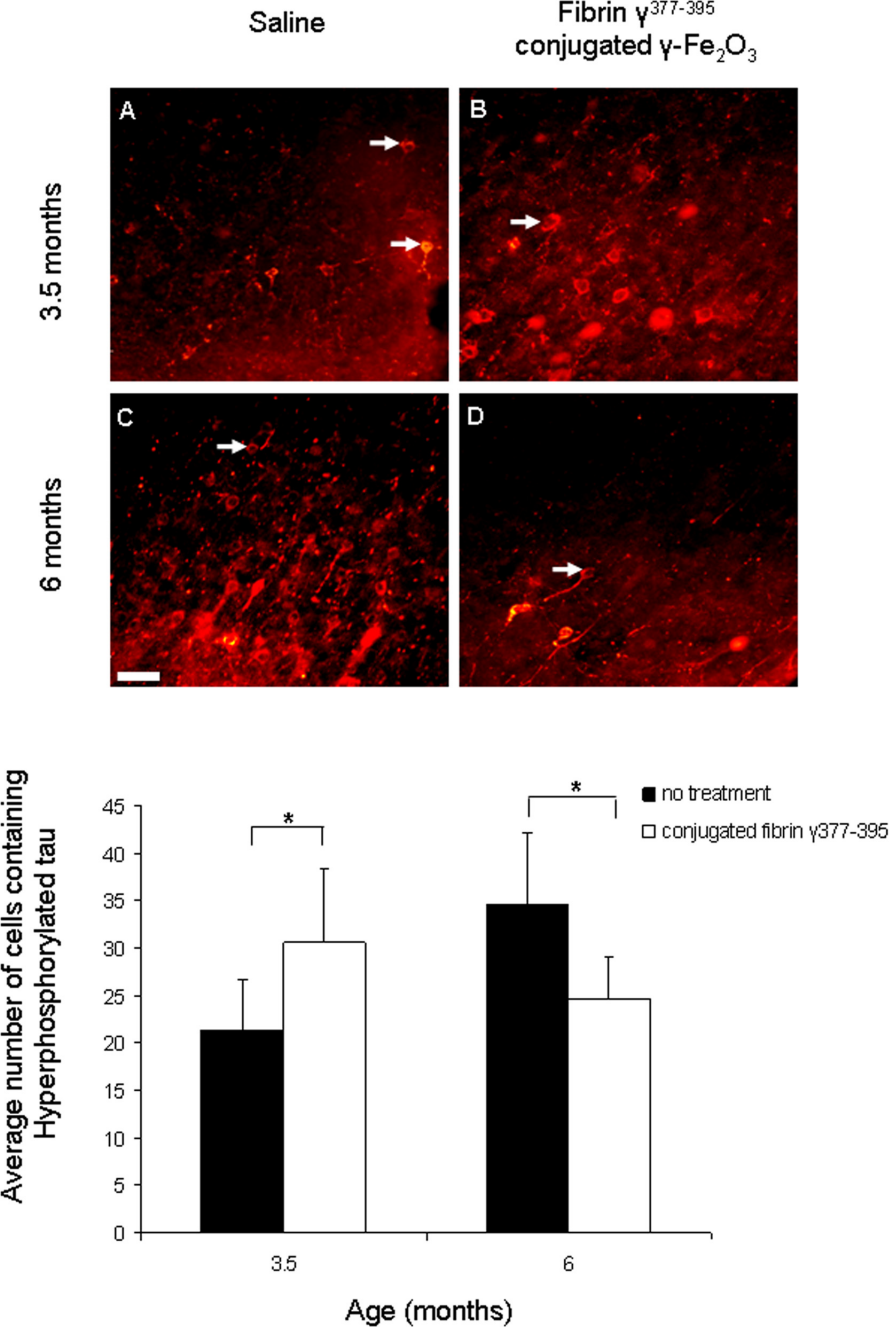
**The effect of microglial inhibition on hyperphosphorylated tau is age dependent.** Staining of hyperphosphorylated tau by AT8 revealed significant differences in neurons containing hyperphosphorylated tau. At age 3.5 months injecting the conjugated fibrin γ^377-395^ peptide **(B)** led to an increase in the number of cells containing hyperphosphorylated tau compared with control **(A)**, (n = 5 mice, 160 areas). In contrast, at age 6 months, a decrease in the number of hyperphosphorylated tau was observed when comparing the conjugated fibrin γ^377-395^ peptide **(D)** with control **(C)**, (n = 6 mice, 133 areas). Scale bar: 50 μm.

After establishing a practical method of delivery for the inactivation of microglia in the transgenic mice model, we sought to determine whether microglial inhibition affected the accumulation of hyperphosphorylated tau in neocortical neurons. After injecting the fibrin γ^377-395^ peptide-conjugated γ-Fe_2_O_3_ nanoparticles, the average hyperphosphorylated tau per area surrounding the site of injection was quantified using AT8 antibody staining (Figure 5). Overall, no significant differences were found in the number of cells containing hyperphosphorylated tau (white arrows) in the fibrin-injected brain areas when compared with those in saline-injected control hemispheres (F = 0.05;df = 1,289;NS). A significant difference was found for age (F = 4.22; df = 1,289; p ≤ 0.05). A significant interaction effect was found between age and treatment: (F = 29.5; df = 1,289; p ≤ 0.05), revealing an opposing effect between the “young” and “old” mice following microglial inhibition on the accumulation of hyperphosphorylated tau in cortical neurons. Compared to controls, at age 3.5 months, an increase in the number of cells containing hyperphosphorylated tau was found following microglial inhibition, whereas at age 6 months a decrease in the number of cells containing hyperphosphorylated tau was observed. The results are summarized in the graph in Figure 5. These results suggest that the involvement of microglia in the development of hyperphosphorylated tau is age-dependent or alternatively, dependent on the stage of pathological tau accumulation.

**Figure 6:**
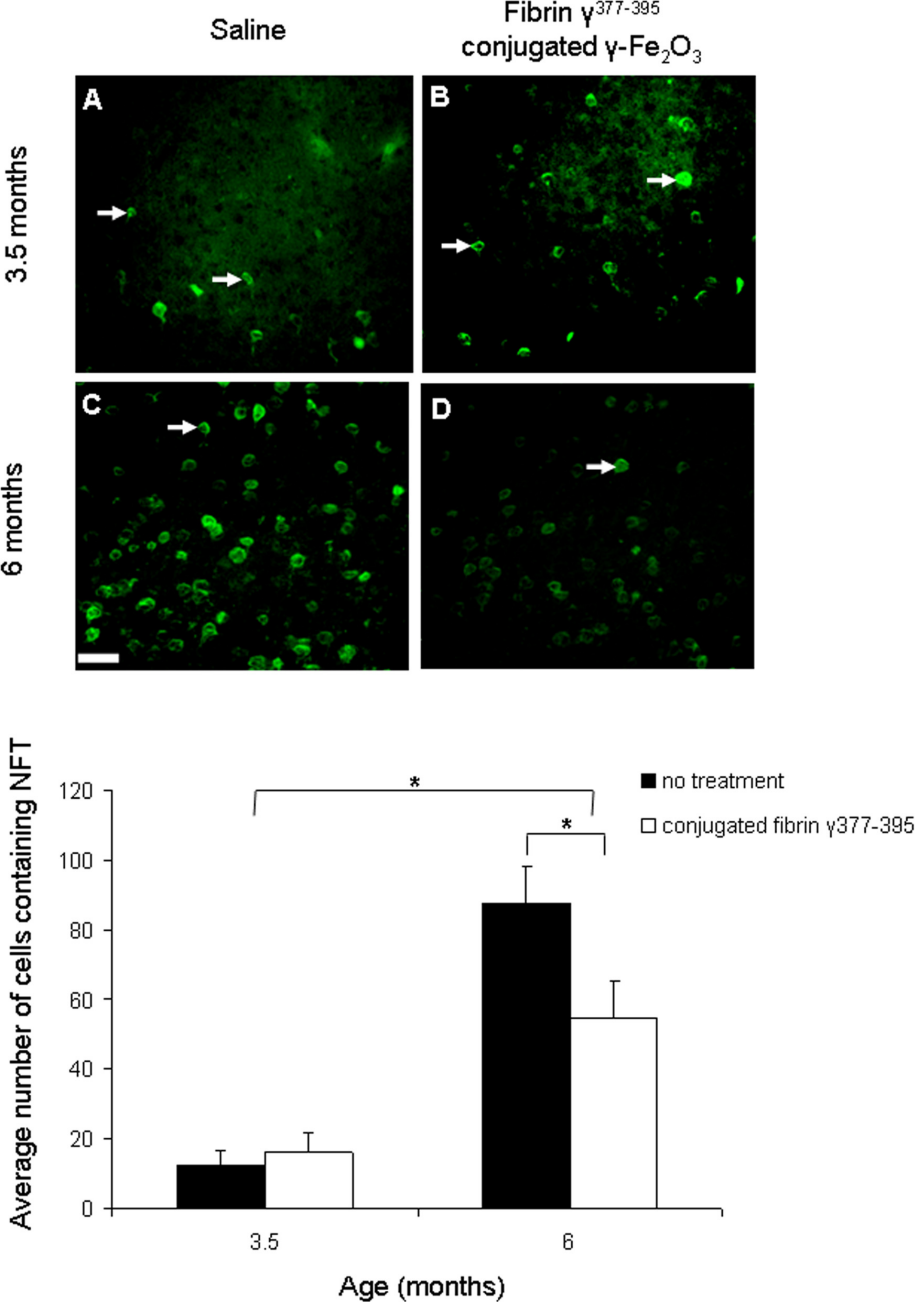
**The effect of microglial inhibition on NFTs.** Staining of NFTs by Thioflavin-S revealed a reduction in neurons containing NFT in mice aged 6 months when comparing saline **(C)** with fibrin γ^377-395^ peptide-conjugated γ-Fe_2_O_3_ nanoparticles **(D)** (n = 8 mice, 124 areas). No differences in NFTs were found in mice aged 3.5 months when comparing saline **(A)** with the treatment hemisphere **(B)** (n = 4 mice, 246 areas). Scale bar: 50 μm.

We then tested the effects of microglial inhibition on the number of tangle-containing neurons. The effect of the fibrin γ^377-395^ peptide-conjugated γ-Fe_2_O_3_ nanoparticles on tangle formation in the frontal cortex is shown in Figure 6. The results are similar to those for the hyperphosphorylated tau. A significant increase is found in the number of tangle-containing neurons (white arrows) with age (F = 860; df = 1,366; p ≤ 0.05). The effect of microglial inhibition is significant overall (F = 88.96; df = 1,366; p ≤ 0.05). Again, a significant interaction between age and treatment was found: (F = 136; df = 1,366; p ≤ 0.05). Post hoc comparisons revealed that the differences in tangle-containing neurons between control and treated animals at 3.5 months were non significant, which is not surprising given the very low number of tangles occurring in neocortex at this age in the model. The significant reduction in tangle-containing neurons between control and treated animals occurred only at the age at which significant tangle aggregation occurs in this model. The results are summarized in the graph in Figure 6.

Taken together, these results reveal that microglial inhibition reduces both hyperphosphorylated tau-containing neurons and tangle-containing neurons in older mice. However, hyperphosphorylated tau-containing neurons are increased in younger mice, indicating that the effects of microglial inhibition depend on the stage of the pathological accumulation of tau.

## Discussion

The role of microglia cells in tau pathology is still an open question, and the literature is filled with seemingly contradictive findings on this subject. Previous studies [21,42] have shown that dystrophic rather than activated microglial cells are associated with tau pathology. On the other hand, other studies described that chronic inflammation, caused by a high level of microglial activation, may exacerbate neurodegeneration [26,43]. For example, Yoshiyama et al. [24] have recently shown that administering immunosuppressors reduced tau pathology when mice were treated at an early age for a period of over 8 months.

In light of these contrasting findings we decided to specifically inhibit the activation of microglia cells to rule out systemic factors arising from immunosuppressor treatments. The inhibition was facilitated using fibrin γ^377-395^ peptide-conjugated γ-Fe_2_O_3_ nanoparticles, which significantly decreased the number of activated microglial cells as compared to the administration of the free peptide in both age groups examined. This observation was enabled by conjugating of fibrin γ^377-395^ peptide to the γ-Fe_2_O_3_ nanoparticles, which may have increased the stability of the peptide against proteolytic enzymes present in the body, thus prolonging its activity, compared to that of the free peptide. A similar stabilization effect was reported previously for thrombin [39], glial-cell derived neurotrophic factor [40], bFGF [36], TRAIL [37], factor VIIa [41], and methotrexate [38] conjugated to γ-Fe_2_O_3_ nanoparticles.

Our results indicate that the stage of pathology may affect microglial function in relation to the disease process, revealed by the interaction between age and treatment for hyperphosphorylated tau, where an inverse relationship between the number of hyperphosphorylated tau and age of treated mice was reported. In “young” mice an increase in the number of cells containing hypersphosphorylated tau was found, whereas in “old” mice a significant decrease in hyperphosphorylated tau containing neurons was observed. These findings suggest that the microglial cells possess both a beneficial and a harmful role in the pathology of tau depending on the stage of the pathology. We postulate that this effect is a result of two different stages of the pathology: the early stage, when the microglial cells attempt to remove cellular debris before further damage can occur [44] and when they release neurotrophic factors such as NGF, NT-3 and BDNF which have been demonstrate to be neuroprotective [23]; and the late stage of the pathology, when the microglia cells are over activated and excessive levels of pro-inflammatory cytokines are released [45]. The cytokines secreted mainly by activated microglia, namely, interleukine-1 (IL-1), TNF-α, and interleukin-6 (IL-6), could affect the normal behavior of neurons and exacerbate tau pathology [46]. Several studies have described that IL-1 [43,47] and IL-6 [46] can induce phosphorylation of tau in neuronal cells, further strengthening this suggestion.

In “young” mice, our study found no differences in NFTs count between mice treated with the fibrin γ^377-395^ peptide-conjugated γ-Fe_2_O_3_ nanoparticles and the control, whereas in “old” mice a significant reduction in the number of cells containing NFTs was observed. This significant finding is in accordance with a previous study [48] which described that tau is first hypersphosphorylated and then aggregated into NFTs at a later stage, rather than hyperphosphorylated tau accumulation and tangle aggregation occurring in separate populations of cells.

## Conclusions

This study has shown that microglial cells posses a complex relationship with neuropathology. Our work demonstrates that microglial cells can be an effective and perhaps necessary target for therapeutic strategies. Similar evidence has been found in mouse models of amyloid-β pathology [49,50]. Depending on the stage of the disease, it may be necessary to use a differential approach to microglial activation: increasing activation at early stages and reducing activation at later stages. This has important implications for immunotherapeutic approaches to many neurodegenerative diseases in addition to tauopathies.

This study has also shown the efficacy of γ-Fe_2_O_3_ nanoparticles in the stable delivery of fibrin γ^377-395^ peptide to nervous tissue in vivo. This method can be used for the study of delivery of a number of substances to the brain, as well as monitoring their presence over time. In addition to use as a tool for experimental drug delivery, it may also be possible to use γ-Fe_2_O_3_ nanoparticles as a therapeutic approach for controlled release of substances in the central nervous system.

## Materials and methods

### Synthesis of bioactive γ-Fe_2_O_3_ nanoparticles

The following analytical-grade chemicals were purchased from commercial sources and used without further purification: ferrous chloride tetrahydrate, hydrochloric acid (1 M), sodium hydroxide (1 M standard solution), sodium chloride, sodium nitrite, gelatin from porcine skin, divinyl sulfone (DVS), triethylamine (TEA) from Sigma (Israel); fibrinogen-derived γ^377-395^ peptide from AnaSpec (Israel); Bicarbonate buffer (BB, 0.1 M, pH 8.4) and phosphate-buffered saline (PBS free of Ca^+2^ and Mg^+2^, 0.1M, pH 7.4) from Biological-Industries (Israel); midi MACS magnetic columns from Miltenyi Biotec GmbH (Germany). Water was purified by passing deionized water through an Elgastat Spectrum reverse osmosis system (Elga, High Wycombe, UK).

γ-Fe_2_O_3_ and DVS-derivatized γ-Fe_2_O_3_ nanoparticles were prepared according to our previous publications [36]. The covalent conjugation of fibrin γ^377-395^ peptide to the γ-Fe_2_O_3_ nanoparticles was accomplished via the Michael addition reaction [36]. Briefly, 50 μl of a fibrin γ^377-395^ peptide PBS solution (1 mg/ml, pH 7.4) were added to 143 μl of the DVS-derivatized γ-Fe_2_O_3_ nanoparticles dispersed in BB (3.5 mg/ml, pH 8.4) at a [nanoparticles]/[γ^377-395^] weight ratio of 10. The reaction mixture was then shaken at room temperature for 18 h. Blocking of the residual double bonds was then accomplished by adding 1% glycine (w/v) and then shaking for an additional hour. The obtained fibrin γ^377-395^ peptide-conjugated γ-Fe_2_O_3_ nanoparticles were than washed from non-magnetic waste with PBS using the high gradient magnetic field (HGMF) technique [34]. The concentration of the γ^377-395^ peptide conjugated to the nanoparticles was determined by measuring the unbound peptide using the Bradford assay and subtracting it from the initial concentration [51].

### Animal model

rTg4510 mice were used in this study. Santa Cruz et al. [52] created these transgenic mice using a CaMII alpha promoter driven by a tetracycline operator to focus human mutant P301L tau over-expression in the forebrain (hippocampus and higher cortical layers) of the mice.

Nine rTg4510 mice aged 3.5 months (“young”) and 7 rTg4510 aged 6 months (“old”) were used in this study. Mice weight, on average, was 25g. All procedures were performed in accordance with the NIH Guide for the Care and Use of Laboratory Animals, and with the Bar-Ilan University guidelines for the use and care of laboratory animals in research, approved and supervised by the Institutional Animal Care and Use Committee.

### Intracranial injections

3.5 and 6 month old rTg4510 mice were injected intracranially with the fibrin γ^377-395^ peptide-conjugated γ-Fe_2_O_3_ nanoparticles and free fibrin γ^377-395^ peptide (1:3 weight ratio). Briefly, mice were anaesthetized before surgery with a ketamine:xylazine at 150 and 12 mg/kg, respectively [53]. The animals were then placed into a stereotaxic apparatus with a heating pad to maintain body temperature. Stereotaxic coordinates were bregma + 1.5 mm anterior-posterior, 1.5 mm lateral and 1.8 mm vertical for frontal cortex. Subsequently, 10 μL of the fibrin γ^377-395^ peptide-conjugated γ-Fe_2_O_3_ nanoparticles dispersed in PBS (2 mg/ml, 10 ug bound fibrin γ^377-395^ per injection) and free fibrin γ^377-395^ peptide dissolved in a PBS solution (3 mg/ml), were intracranially injected in the right and left hemispheres, respectively. The injection holes were then filled with bone wax. The mice were stitched and then returned to their cages. All mice used in our experiments were then sacrificed 30 days post injection via an intracardial perfusion described in the next section.

### Tissue preparation

The animals were given a lethal dose of thiopental sodium solution and were perfused intracardially with isotonic PBS (pH 7.3) followed by 4% paraformaldehyde solution in 0.1M PBS. Once the perfusions were complete, the brains were rapidly removed from the skulls and were placed in a 4% paraformaldehyde solution for post-fixation of at least 24 h at 4°C. After 24 h, the brains were transferred to a 30% sucrose solution for two days. The brains were then frozen in dry ice and placed in −80°C over night. Before sectioning the brains with a cryostat, the brains were covered using an optimal cutting temperature (OCT) solution and were placed in −20°C. Once the OCT solidified, the brains were placed in the cryostat with the frontal side up. Tissue samples were selected from areas of cortex adjacent to the injection sites. The brains were sliced to 25 μm coronal sections. Each sample consisted of 5 slices. Controls were the contralateral areas. Sampling within the slices was performed at 3 sites, each 400 × 340 μm. The brain sections were then individually picked up using a paint brush and were transferred to a well filled with 0.1M PBS. Slices were then either stained immediately following the appropriate protocol or stored in a cryoprotectant solution at −20°C until further use.

### Staining methods

#### Lectin staining

The slices were first introduced into a PBS solution in order to remove the OCT from the tissue. The slices were treated with FITC-conjugated Bandeiraea simplicifolia isolectin B4 (IB-4; 1:50; Sigma-Aldrich) for 1 h, and then were washed three times for 5 min each in PBS. The slices were then mounted on slides and stored in the dark to air-dry. A drop of DAPI (1:2000; Sigma-Aldrich) solution was added to sections after drying using a Pasteur pipette for 1 min and washed twice for 1 min each in PBS. The slides were then sealed using a cover slip.

#### Iron staining

The slices were mounted on slides and placed in 2% potassium ferrocyanide solution with 2% HCl solution for 30 min. Afterwards, the slides were washed three times in distilled water and sealed with a cover slip.

#### Immunohistochemistry

The slices were first introduced into a PBS solution in order to remove the OCT from the tissue. The slices were incubated in 0.5% Triton for 20 min and then washed three times for 5 min each in PBS. The slices were then incubated in a blocking solution (NGS, 5% in PBS; from Jackson Immuno Research) for 1 h. After the blocking stage, slices were treated with Anti-Human-PHF-Tau Monoclonal Antibody (AT8; 1:1000 in normal goat serum; Thermo Scientific) and then left overnight at 4°C. Following incubation, the slices were washed three times for 5 min each in PBS. The slices were treated with Cy3 donkey anti-mouse (Cy3 1:500, Jackson Immuno Research). After 1 h, the slices were washed three times in PBS. The slices were then mounted on slides, stored in the dark to air-dry and then sealed using a cover slip. If NFTs staining was performed as well, a drop of 0.05% Thio-S solution (Sigma-Aldrich) was added to sections after drying using a Pasteur pipette. The sections were then incubated for 8 min in the dark, followed by two washes for 10 sec in 80% ethanol solution and one wash in distilled water. The slides were then stored in the dark to air-dry and sealed using a cover slip.

To study microglia morphology, Iba-1 staining was performed. The endogenous peroxidase was blocked by 10% H2O2, 0.01% Triton X-100 in PBS, and 3% Methanol. After 30 minutes blocking was performed by 20% NGS for 1 h. Slices were then incubated overnight in rabbit polyclonal anti-IBA1 antibody (1:250; Wako Chemicals) with 2% NGS. Following incubation the sections were treated with a secondary antibody, Anti rabbit IgG Peroxidase (1:200; Santa Cruz) for 1 h followed by incubation with ABC solution (Vector) for 30 minutes. The peroxidase labeling was visualized by incubation with commercial DAB Substrate Kit (Vector) for a few minutes. The reaction was stopped by placing the slices in PBS. Slices were then mounted, dehydrated, and cover-slipped.

### Statistical analysis

Two to three different areas surrounding the site of injection from five slices from each mouse were defined for the analysis of activated microglial cells and cells containing hypersphosphorylated tau and NFTs. Statistical analysis was performed using 2-way-ANOVAs followed by Tukey multiple comparison tests. A significance level of p ≤ 0.05 was set for all statistical tests.

## Competing interests

The authors declare that they have no competing interests.

## Authors’ contributions

MG participated in the design of the study, conducted the experiments, analyzed the data, and wrote the manuscript. HS participated in the design of the study, synthesized the bioactive nanoparticles and helped to draft the manuscript. NMC conducted the experiments. SM and EAS conceived the idea of the project, SM supervised the synthesis of the bioactive nanoparticles, and EAS designed and supervised the biological experiments, analysis and the writing of the manuscript. All authors read and approved the final manuscript. All authors have reviewed the manuscript.

## References

[B1] Spillantini MG Goedert M Tau protein pathology in neurodegenerative diseases Trends Neurosci 1998 21 428 433 10.1016/S0166-2236(98)01337-X 9786340

[B2] Weingarten MD Lockwood AH Hwo SY Kirschner MW A protein factor essential for microtubule assembly Proc Natl Acad Sci U S A 1975 72 1858 1862 10.1073/pnas.72.5.1858 1057175 PMC432646

[B3] Drubin D Kobayashi S Kirschner M Association of tau protein with microtubules in living cells Ann N Y Acad Sci 1986 466 257 268 10.1111/j.1749-6632.1986.tb38398.x 2873777

[B4] Neve RL Harris P Kosik KS Kurnit DM Donlon TA Identification of cDNA clones for the human microtubule-associated protein tau and chromosomal localization of the genes for tau and microtubule-associated protein 2 Brain Res 1986 387 271 280 3103857 10.1016/0169-328x(86)90033-1

[B5] Goedert M Spillantini MG Jakes R Rutherford D Crowther RA Multiple isoforms of human microtubule-associated protein tau: sequences and localization in neurofibrillary tangles of Alzheimer’s disease Neuron 1989 3 519 526 10.1016/0896-6273(89)90210-9 2484340

[B6] Kosik KS The molecular and cellular pathology of Alzheimer neurofibrillary lesions J Gerontol 1989 44 55 58 10.1093/geronj/44.6.55 2497170

[B7] Arrasate M Perez M Avila J Tau dephosphorylation at tau-1 site correlates with its association to cell membrane Neurochem Res 2000 25 43 50 10.1023/A:1007583214722 10685603

[B8] Kawamata T Taniguchi T Mukai H Kitagawa M Hashimoto T Maeda K Ono Y Tanaka C A protein kinase, PKN, accumulates in Alzheimer neurofibrillary tangles and associated endoplasmic reticulum-derived vesicles and phosphorylates tau protein J Neurosci 1998 18 7402 7410 9736660 10.1523/JNEUROSCI.18-18-07402.1998 PMC6793236

[B9] Alvarez A Toro R Caceres A Maccioni RB Inhibition of tau phosphorylating protein kinase cdk5 prevents beta-amyloid-induced neuronal death FEBS Lett 1999 459 421 426 10.1016/S0014-5793(99)01279-X 10526177

[B10] Zhang YJ Xu YF Liu YH Yin J Wang JZ Nitric oxide induces tau hyperphosphorylation via glycogen synthase kinase-3beta activation FEBS Lett 2005 579 6230 6236 10.1016/j.febslet.2005.09.095 16253246

[B11] Lee HG Perry G Moreira PI Garrett MR Liu Q Zhu X Takeda A Nunomura A Smith MA Tau phosphorylation in Alzheimer’s disease: pathogen or protector? Trends Mol Med 2005 11 164 169 10.1016/j.molmed.2005.02.008 15823754

[B12] Spires TL Orne JD SantaCruz K Pitstick R Carlson GA Ashe KH Hyman BT Region-specific dissociation of neuronal loss and neurofibrillary pathology in a mouse model of tauopathy Am J Pathol 2006 168 1598 1607 10.2353/ajpath.2006.050840 16651626 PMC1606598

[B13] Kreutzberg GW Microglia: a sensor for pathological events in the CNS Trends Neurosci 1996 19 312 318 10.1016/0166-2236(96)10049-7 8843599

[B14] Gonzalez-Scarano F Baltuch G Microglia as mediators of inflammatory and degenerative diseases Annu Rev Neurosci 1999 22 219 240 10.1146/annurev.neuro.22.1.219 10202538

[B15] Thomas WE Brain macrophages: evaluation of microglia and their functions Brain Res Brain Res Rev 1992 17 61 74 10.1016/0165-0173(92)90007-9 1638276

[B16] Aloisi F Immune function of microglia Glia 2001 36 165 179 10.1002/glia.1106 11596125

[B17] Hanisch UK Microglia as a source and target of cytokines Glia 2002 40 140 155 10.1002/glia.10161 12379902

[B18] Cullheim S Thams S The microglial networks of the brain and their role in neuronal network plasticity after lesion Brain Res Rev 2007 55 89 96 10.1016/j.brainresrev.2007.03.012 17509690

[B19] Trapp BD Wujek JR Criste GA Jalabi W Yin X Kidd GJ Stohlman S Ransohoff R Evidence for synaptic stripping by cortical microglia Glia 2007 55 360 368 10.1002/glia.20462 17136771

[B20] Neumann H Kotter MR Franklin RJ Debris clearance by microglia: an essential link between degeneration and regeneration Brain 2009 132 288 295 18567623 10.1093/brain/awn109 PMC2640215

[B21] Streit WJ Braak H Xue QS Bechmann I Dystrophic (senescent) rather than activated microglial cells are associated with tau pathology and likely precede neurodegeneration in Alzheimer’s disease Acta Neuropathol 2009 118 475 485 10.1007/s00401-009-0556-6 19513731 PMC2737117

[B22] Li L Lu J Tay SS Moochhala SM He BP The function of microglia, either neuroprotection or neurotoxicity, is determined by the equilibrium among factors released from activated microglia in vitro Brain Res 2007 1159 8 17 17572395 10.1016/j.brainres.2007.04.066

[B23] Nakajima K Honda S Tohyama Y Imai Y Kohsaka S Kurihara T Neurotrophin secretion from cultured microglia J Neurosci Res 2001 65 322 331 10.1002/jnr.1157 11494368

[B24] Yoshiyama Y Higuchi M Zhang B Huang SM Iwata N Saido TC Maeda J Suhara T Trojanowski JQ Lee VM Synapse loss and microglial activation precede tangles in a P301S tauopathy mouse model Neuron 2007 53 337 351 10.1016/j.neuron.2007.01.010 17270732

[B25] Gorlovoy P Larionov S Pham TT Neumann H Accumulation of tau induced in neurites by microglial proinflammatory mediators FASEB J 2009 23 2502 2513 10.1096/fj.08-123877 19289607

[B26] Kitazawa M Oddo S Yamasaki TR Green KN LaFerla FM Lipopolysaccharide-induced inflammation exacerbates tau pathology by a cyclin-dependent kinase 5-mediated pathway in a transgenic model of Alzheimer’s disease J Neurosci 2005 25 8843 8853 10.1523/JNEUROSCI.2868-05.2005 16192374 PMC6725603

[B27] Ugarova TP Solovjov DA Zhang L Loukinov DI Yee VC Medved LV Plow EF Identification of a novel recognition sequence for integrin alphaM beta2 within the gamma-chain of fibrinogen J Biol Chem 1998 273 22519 22527 10.1074/jbc.273.35.22519 9712878

[B28] Adams RA Bauer J Flick MJ Sikorski SL Nuriel T Lassmann H Degen JL Akassoglou K The fibrin-derived gamma377-395 peptide inhibits microglia activation and suppresses relapsing paralysis in central nervous system autoimmune disease J Exp Med 2007 204 571 582 10.1084/jem.20061931 17339406 PMC2137908

[B29] Slomkowski S Gosecki M Progress in nanoparticulate systems for peptide, proteins and nucleic acid drug delivery Curr Pharm Biotechnol 2011 12 1823 1839 10.2174/138920111798377003 21902630

[B30] de Vries IJM Lesterhuis WJ Barentsz JO Verdijk P van Krieken JH Boerman OC Oyen WJG Bonenkamp JJ Boezeman JB Adema GJ Magnetic resonance tracking of dendritic cells in melanoma patients for monitoring of cellular therapy Nat Biotechnol 2005 23 1407 1413 10.1038/nbt1154 16258544

[B31] Hergt R Hiergeist R Hilger I Kaiser WA Lapatnikov Y Margel S Richter U Maghemite nanoparticles with very high AC-losses for application in RF-magnetic hyperthermia J Magn Magn Mater 2004 270 345 357 10.1016/j.jmmm.2003.09.001

[B32] Scherer F Anton M Schillinger U Henke J Bergemann C Kruger A Gansbacher B Plank C Magnetofection: enhancing and targeting gene delivery by magnetic force in vitro and in vivo Gene Ther 2002 9 102 109 10.1038/sj.gt.3301624 11857068

[B33] Rudge SR Kurtz TL Vessely CR Catterall LG Williamson DL Preparation, characterization, and performance of magnetic iron-carbon composite microparticles for chemotherapy Biomaterials 2000 21 1411 1420 10.1016/S0142-9612(00)00006-5 10872770

[B34] Margel S Gura S Nucleation and growth magnetic metal oxide nanoparticles and its use Israel patent 2006 No. WO9962079

[B35] Perlstein B Ram Z Daniels D Ocherashvilli A Roth Y Margel S Mardor Y Convection-enhanced delivery of maghemite nanoparticles: increased efficacy and MRI monitoring Neuro Oncol 2008 10 153 161 10.1215/15228517-2008-002 18316474 PMC2613817

[B36] Skaat H Ziv O Shahar A Margel S Enhancement of the growth and differentiation of nasal olfactory mucosa cells by the conjugation of growth factors to functional nanoparticles Bioconjugate Chem 2011 22 2600 2610 10.1021/bc200454k 22029397

[B37] Margel S Perlstein B Brodie C Polymer nanoparticles coated by magnetic metal oxide and uses thereof US patent 2009 No. WO 2009/040811

[B38] Corem-Salkmon E Ram Z Daniels D Perlstein B Last D Salomon S Tamar G Shneor R Guez D Margel S Convection-enhanced delivery of methotrexate-loaded maghemite nanoparticles Int J Nanomedicine 2011 6 1595 1602 21904449 10.2147/IJN.S23025 PMC3160945

[B39] Ziv-Polat O Topaz M Brosh T Margel S Enhancement of incisional wound healing by thrombin conjugated iron oxide nanoparticles Biomaterials 2010 31 741 747 10.1016/j.biomaterials.2009.09.093 19850336

[B40] Green-Sadan T Kuttner Y Lublin-Tennenbaum T Kinor N Boguslavsky Y Margel S Yadid G Glial cell line-derived neurotrophic factor-conjugated nanoparticles suppress acquisition of cocaine self-administration in rats Exp Neurol 2005 194 97 105 10.1016/j.expneurol.2005.01.020 15899247

[B41] Shafir G Galperin A Margel S Synthesis and characterization of recombinant factor VIIa-conjugated magnetic iron oxide nanoparticles for hemophilia treatment J Biomed Mater Res Part A 2009 91 1056 1064 10.1002/jbm.a.32296 19107792

[B42] Streit WJ Microglia and neuroprotection: implications for Alzheimer’s disease Brain Res Brain Res Rev 2005 48 234 239 10.1016/j.brainresrev.2004.12.013 15850662

[B43] Li Y Liu L Barger SW Griffin WS Interleukin-1 mediates pathological effects of microglia on tau phosphorylation and on synaptophysin synthesis in cortical neurons through a p38-MAPK pathway J Neurosci 2003 23 1605 1611 12629164 10.1523/JNEUROSCI.23-05-01605.2003 PMC3833596

[B44] Rogers J Strohmeyer R Kovelowski CJ Li R Microglia and inflammatory mechanisms in the clearance of amyloid beta peptide Glia 2002 40 260 269 10.1002/glia.10153 12379913

[B45] Morales I Farias G Maccioni RB Neuroimmunomodulation in the pathogenesis of Alzheimer’s disease Neuroimmunomodulation 2010 17 202 204 10.1159/000258724 20134203

[B46] Quintanilla RA Orellana DI Gonzalez-Billault C Maccioni RB Interleukin-6 induces Alzheimer-type phosphorylation of tau protein by deregulating the cdk5/p35 pathway Exp Cell Res 2004 295 245 257 10.1016/j.yexcr.2004.01.002 15051507

[B47] Mrak RE Griffin WS Potential inflammatory biomarkers in Alzheimer’s disease J Alzheimers Dis 2005 8 369 375 16556968 10.3233/jad-2005-8406

[B48] Avila J Lucas JJ Perez M Hernandez F Role of tau protein in both physiological and pathological conditions Physiol Rev 2004 84 361 384 10.1152/physrev.00024.2003 15044677

[B49] Garcia-Alloza M Ferrara BJ Dodwell SA Hickey GA Hyman BT Bacskai BJ A limited role for microglia in antibody mediated plaque clearance in APP mice Neurobiol Dis 2007 28 286 292 10.1016/j.nbd.2007.07.019 17822910 PMC2193669

[B50] Koenigsknecht-Talboo J Meyer-Luehmann M Parsadanian M Garcia-Alloza M Finn MB Hyman BT Bacskai BJ Holtzman DM Rapid microglial response around amyloid pathology after systemic anti-Abeta antibody administration in PDAPP mice J Neurosci 2008 24 14156 14164 19109498 10.1523/JNEUROSCI.4147-08.2008 PMC2743894

[B51] Bradford MM A rapid and sensitive method for the quantitation of microgram quantities of protein utilizing the principle of protein-dye binding Anal Biochem 1976 72 248 254 10.1016/0003-2697(76)90527-3 942051

[B52] Santacruz K Lewis J Spires T Paulson J Kotilinek L Ingelsson M Guimaraes A DeTure M Ramsden M McGowan E Forster C Yue M Orne J Janus C Mariash A Kuskowski M Hyman B Hutton M Ashe KH Tau suppression in a neurodegenerative mouse model improves memory function Science 2005 309 476 481 10.1126/science.1113694 16020737 PMC1574647

[B53] Stern EA Bacskai BJ Hickey GA Attenello FJ Lombardo JA Hyman BT Cortical synaptic integration in vivo is disrupted by amyloid-beta plaques J Neurosci 2004 24 4535 4540 10.1523/JNEUROSCI.0462-04.2004 15140924 PMC6729398

